# Paternal perception of infant sleep risks and safety

**DOI:** 10.1186/s40621-018-0140-4

**Published:** 2018-04-10

**Authors:** Heather M. Hirsch, Samantha H. Mullins, Beverly K. Miller, Mary E. Aitken

**Affiliations:** 1 Center for Applied Research and Evaluation, Department of Pediatrics, University of Arkansas for Medical Sciences, Little Rock, AR 72202 USA; 2 Injury Prevention Center, Arkansas Children’s, Little Rock, AR 72202 USA; 3 Arkansas Children’s Research Institute, Little Rock, AR 72202 USA

**Keywords:** Sudden unexpected infant death (SUID), Sleep, Male caregivers, Barriers, Messaging

## Abstract

**Background:**

Sudden Unexpected Infant Death (SUID) results in 3400 sleep-related deaths yearly in the United States, yet caregivers’ compliance with safe sleep recommendations remains less than optimal. Paternal caregiver’s attitudes toward infant safe sleep messages are largely unaddressed, despite established differences between female and male caregiver perceptions. This study aimed to explore the determinants of safe sleep practices among male caregivers.

**Methods:**

Focus groups were conducted in Arkansas with male caregivers of infants ages 2–12 months to discuss infant sleep routines, parental roles, sources for safe sleep information, and messaging suggestions for safe sleep promotion. The Health Belief Model of behavior change framed a moderator guide. Transcript-based analysis was used, and data were managed using HyperRESEARCH (version 2.8.3). The transcribed data were coded to identify significant themes.

**Results:**

Ten focus groups were conducted with 46 participants. Inconsistent adherence to safe sleep practices was reported. Participants were more likely to describe safe location (57% of participants) and supine position behaviors (42%) than an uncluttered bed environment (26%). Caregivers acknowledged the importance of recommended safe sleep behavior, but admitted to unsafe practices, such as co-sleeping and unsafe daytime sleep. Lack of perceived risk, comfort, and/or resources, and disagreement among family members about safety practices were identified as barriers. Participants voiced concerns that current advertising portrays males as incompetent caregivers. Suggestions included portraying positive images of fathers and male caregivers acting to promote safety and the incorporation of statistics about the hazards of unsafe sleep to better engage fathers. Potential distribution venues included sporting events, home improvement and/or automotive stores, and social media from trusted sites (e.g. hospitals or medical professionals).

**Conclusions:**

Male caregivers demonstrate some knowledge base about infant sleep safety, but are not fully practicing all aspects of safe sleep. Targeted messaging towards male caregivers that includes factual information and statistics along with representing males in a positive light is desired.

## Background

Interventions and promotional campaigns to increase compliance with recommendations from the American Academy of Pediatrics (AAP) to reduce sleep-related deaths in infants have focused primarily on maternal perspectives. Despite research that suggests that fathers’ involvement in caregiving responsibilities may lead to improved infant and maternal sleep 6 months postpartum (Moon et al. 2010), there is a lack of evidence in the literature regarding the knowledge, attitudes, beliefs and behaviors from paternal caregivers on infant sleep (Sadeh et al. 2010; Tikotzky et al. 2015).

There is evidence, however, supporting different perceptions of sleep-related temperament between female and male caregivers (Keener et al. 1988; Millikovsky-Ayalon et al. 2015). Further, research has found that while female caregivers are more likely to be responsible for nighttime sleep behaviors, male caregivers are more likely to contribute to care during “night time waking” (Moon et al. 2010; Tikotzky et al. 2015; Ball et al. 2000; Goodlin-Jones et al. 2001; Tikotzky et al. 2011). This suggests that male caregivers make critical decisions regarding sleep environment and positions for intermittent or short-term sleep of the infant. Some research highlights perspectives on parent-child co-sleeping. In a 1995 study from the United Kingdom, fathers were worried about practicing parent-child co-sleeping for fear of disruption of parental sleep and fear of “squashing” the infant. The majority of these fathers (81%) overcame their initial fears and co-slept, citing perceived benefits of triadic co-sleeping (mother-father-baby) as a method of strengthening the paternal-child relationship (Ball et al. 2000).

The sources from which parents receive safety and parenting advice are important, because they can be influential to caregiving behaviors, including infant sleep practices. The role of social media in promoting peer approval of unsafe sleep behaviors has not been studied, although this is a major source of information for young mothers and may be an appropriate messaging outlet for male caregivers (Holtz et al. 2015; Khanom et al. 2013).

The purpose of this study was to identify key barriers and facilitators to infant safe sleep practices among male caregivers and to gather suggestions for messaging to influence male caregivers in implementing safe sleep practices. The specific objectives were: (1) to determine paternal knowledge of recommendations for sleep of infants; (2) to identify reported and demonstrated sleep practices and behaviors; (3) to explore attitudes and beliefs toward best practice recommendations for safe sleep; and (4) to explore preferences for messaging content and delivery.

## Methods

Focus groups (FGs) have proved useful for gathering rich, qualitative data in past studies related to attitudes toward injury prevention and infant safe sleep (Adams et al. 2013; Herman et al. 2015; Poag et al. 2016; Tong et al. 2007). This study used FGs to identify barriers to and influences of practicing safe sleep for infants by male caregivers. Male caregivers were defined as males who put an infant (2–12 months of age) to sleep (nap or night time) four or more times per week. The study was approved by the University of Arkansas for Medical Sciences Institutional Review Board and was exempt from written informed consent.

### Participant selection and compensation

Recruitment for FGs was performed via passive promotional materials (flyers, social media, and emails) and direct recruitment. Convenience sampling occurred throughout Arkansas at pediatric and community health clinics, public businesses, and via community partners connected with churches and daycares. A standard screening questionnaire was used to determine participant eligibility and facilitate FG scheduling. Exclusion factors included not caring for an infant on a regular basis or inability to speak English. Eligible participants were given reminder phone calls and confirmation emails or text messages prior to the FG session to encourage attendance.

FG compensation included a meal, $40 retail gift card, and infant safe sleep information and resources. Also, any questions pertaining to infant safety were addressed following FG discussion. At the beginning of each session, confidentiality was explained and participants had the right not to answer any question(s) and could leave at any time; no participants left early.

### Framework for moderated discussions

The Health Belief Model (HBM) formed the theoretical basis for the moderator guide used in the focus group discussions for this project (Rosenstock et al. 1988). The HBM proposes that health behavior change will result from two major factors including belief in health risk and the efficacy of a health behavior intervention. In addition to the HBM factors, an assessment of self-efficacy was also included in the moderator guide.

### Data collection and analysis

FGs were conducted by trained project staff, consisting of a moderator and assistant moderator, who had widespread knowledge of FG methodology and extensive experience with injury prevention research. The structured moderator guide was utilized to direct the group conversation using an open-ended format that encouraged diverse perspectives and attitudes. In addition to participating in the moderated group discussion, participants utilized dolls and props to demonstrate their normal infant sleep routine, and rated pictures depicting infant sleep environments as safe, unsafe, or unsure.

The assistant moderator maintained field notes and recorded each FG session for transcription. Following each FG, study staff debriefed to identify initial themes and impressions. Standard methods of qualitative analysis were used to examine the FG data. Transcript-based analysis via the computerized content analysis software, HyperRESEARCH version 3.5.2 (Research Ware Inc 1988) was performed to ensure accurate interpretation of discussions. Transcripts were coded to identify reoccurring major and minor themes amongst FG discussions.

## Results

Ten FG sessions were conducted with 2–11 participants in each session. A total of 46 participants attended with 67% identifying as African American and 33% as white. One participant also identified as Hispanic and/or Latino. Most participants identified themselves as fathers/stepfathers (65%), but grandfathers/uncles/cousins (21%), expectant fathers with caregiving experience (7%) or friends/roommates/etc. (7%) were also present. There was a mixture of caregivers with single or multiple children. Subjects ranged in age from 18 to 67 years old.

Using the HBM framework, development of a coding tree resulted in the identification of five major themes of male caregiver’s knowledge and influences of infant safe sleep: (1) sleep practices and behaviors, (2) knowledge of infant safe sleep, (3) barriers and (4) facilitators to practicing infant safe sleep, and (5) infant sleep safety messaging suggestions. Each theme encompasses multiple subthemes. The remaining sections detail the overall results and representative quotations for each of these key thematic areas.

To identify male caregiver’s infant sleep practices, participants were asked to discuss and/or demonstrate their child’s night time sleep routine. These male caregivers were more likely to describe the use of a safe sleep location (57%) and supine position (42%) than an uncluttered bed environment (26%). Many participants laid their infant on his/her back for sleep, but several laid a young infant (2–6 months old) on its side. Regarding safe sleep location, most participants stated they owned a crib, pack n’ play, or bassinette, but allowed infant sleep on an adult bed, couch, or other unsafe surface. For instance, one participant stated his infant was, “…notorious for not liking the pack ‘n play …for naptime …sometimes she may sleep in there, but for the most part she’ll sleep on us.”

The risky practice of co-sleeping in an adult bed was common among these male caregivers. One participant admitted, “we…sleep with her (infant) some in the bed or on one of our chests,” and others felt that was a safer alternative than co-sleeping on another surface as it “…made us feel a little better about it cause we have a very large king-sized bed, so at least there wasn’t any uh worry of like her falling off the side of the bed cause we’d just get in the middle of it and uh then be good.” Several participants recalled falling asleep with their infants unintentionally: “two or three times I fell asleep with her on my chest on the couch, also. But the last time I did that she did slip out of my arms and fall off the, the couch and I was ALRIGHT! NOPE! Never sleeping on the couch with her, only on the bed.”

Most participants using safe locations such as cribs did not keep the areas safe from clutter and other risks. They often used blankets to swaddle or cover infants and allowed soft items (i.e., stuffed animals and pillows) in the environment. Most infants were dressed appropriately in less than three layers, but many were additionally swaddled with a blanket. Some male caregivers utilized sleep sacks, a safe alternative to swaddling. Very few caregivers used other protective factors such as pacifiers.

Many caregivers demonstrated a large disconnect between nap and night sleep. Poor sleep hygiene was apparent as many participants described lack of scheduling and variable duration of naps. For naps in particular, improper location (e.g., couches, adult beds, the floor, or atop of sleeping parents) was common, and even resulted in reports of infants falling off surfaces: “I went to sleep on the couch and he was laying up on my chest and all of a sudden, something went boom, he was on the floor, and I know that this is not the safest way to go to sleep…You can hold them if he’s sleeping like that, but don’t you go too.”

Few participants practiced safe sleep behaviors during nap time, citing a belief that naps are short term with a low risk of harm. One father described their belief in the difference between nap and night time sleep as, “… the placement. I feel like, I wouldn’t let the baby sleep in the swing or bassinette… for nighttime, but a nap, maybe, yeah.” Another caregiver expressed that the expected duration of sleep was an influencing factor, “But like at night, you know they’re gonna sleep for some hours, so you gotta lay them comfortable so you know they’re gonna be good.”

### Knowledge of safe sleep recommendations

Some participants related hearing prior safe sleep messages, while others had little exposure to this information. The majority knew at least one aspect of safe sleep messaging regarding position, location, and dress. Several restated positioning messages: “Don’t put them on their face (stomach),” and “…never on the belly, always on the back.” A few participants indicated the use of a “crib, mattress, and fitted sheet,” as safe locations, and some were aware that “sleep sacks” were appropriate infant dress. Knowledge that was lacking included information on appropriate sleep environments and risks of co-sleeping.

To gauge knowledge qualitatively, participants were asked to rate pictures of sleeping infants as safe, unsafe, or not sure. Approximately 67% of caregivers correctly categorized pictures illustrating unsafe sleep practices such as prone sleep position, unsafe surfaces, or cluttered sleep environments. Participants were unsure of the appropriate slat width of cribs portrayed in the photos. Although the slats of both cribs were safe, participants identified the crib with alternating wider slats as safer than a crib with consistent slat widths (91% vs. 66%, respectively). Substantial discussion about a picture of an infant sleeping in a “Boppy”® pillow occurred, with confusion expressed about objects that may be considered safe for infants when awake but unsafe for sleeping. Also, many participants believed that a few objects, as opposed to many objects, in the crib was safe. When shown images of male caregivers co-sleeping with their infant, many participants agreed the situation was unsafe, but admitted to having unintentionally fallen asleep with their infants in similar circumstances.

### Barriers

Researchers determined barriers preventing male caregivers from practicing infant safe sleep. Lack of knowledge of best safe sleep practice was apparent and thus a major barrier. Other barriers included misunderstandings of infant physiology and the risks of co-sleeping and other unsafe behaviors. These barriers influenced beliefs about sleep risks including susceptibility to sleep-related injuries and potentially fatal outcomes.

Unsafe sleep behaviors reported by the fathers reflected these misunderstandings. For example, some participants didn’t understand the physiology behind an infant’s breathing mechanism, and thus, did not realize that a pillow is a hazard rather than a comforting item: “…we started laying her in there without a pillow, but she wouldn’t sleep, so we added a uh fluffy pillow, and after she would sleep....” Many reasoned their infants needed blankets in their sleep environments to keep warm and allowed their continued use: “…I’ve never seen [the blanket] up over his head or seen it around him so it didn’t bother me...We’ve never had no trouble with it.”

In some cases, caregivers did not perceive risk of harm to their infant in the sleep environment or felt that they had acted to reduce risks. For example, several participants engaged in co-sleeping because they believed their infant was far enough away that they would not roll on top of them: “…we know that me and my wife, we don’t move when we sleep, when we pass out we kinda wake up in the same position… so it’s never been an issue as to we’re scared of rolling over on the child….” Participants engaging in co-sleeping justified the practice with the construct of preventing “crib death” by avoiding the crib entirely. Others advocated incorrect positioning (placing on side) to prevent choking. At least two participants indicated they could not prevent SIDS because it is “…one of them things you ain’t got no control over. There’s nothing you can do about it.” Another stated: “…clearly it’s not making babies die all the time or humans wouldn’t still be here…reassure yourself with that and you feel a little bit better.”

Another barrier included conflicting influences, which were defined by participants as a variety of sources that provide incorrect information regarding infant safe sleep. These sources included challenges stemming from parental disagreement where the infant’s mother wanted to practice an unsafe sleep behavior while the male caregiver did not. Further, older family member’s incorrect advice often influenced parental decisions resulting in unsafe sleeping behaviors. If the caregiver raised a child many years ago, that information often determined current behavior, and many times, the older information was incorrect and outdated. Examples of outdated behavior reported included placing the infant on his side to sleep to prevent reflux, over-bundling an infant to “keep warm”, and putting the infant to bed with a bottle.

Cultural norms were also expressed as influencing factors regarding safe sleep, most notably regarding attitudes and beliefs about a lack of perceived susceptibility to sleep-related deaths. “I’m more country, home remedy, we gonna let God take course” suggested that parents do not believe they can reduce risks. Some participants believed that the use of separate sleep surfaces would negatively impact the relationship between parents and child: “A crib is not a good idea for raising kids...it doesn’t bring a close relationship with the child, mom, and dad.”

Some participants also described attempts to mitigate some risks associated with an infant sleeping on an adult bed. One participant described moving a bed against a wall so that the baby would not roll off. Another participant stated he had no concern about co-sleeping saying, “…she’ll sleep in between my arms, so this kinda would be a cradle.”

Participants cited further negative influence from home products and other industries seen as misleading consumers into buying unnecessary and unsafe products for infants. For example, many participants spoke of infant propping pillows being bought or given as gifts and used as a place for infant sleep despite cases of infants dying in these unsafe sleep environments: “…some of those Boppy® things have actually been recalled to be taken off the market because babies were dying in them, they would roll over and suffocate on them.” Another participant in the same group expressed difficulty in knowing which products had been recalled: “…they don’t put out there and, like, you have to research it.” Although not stated explicitly, some participants believed that the industry still makes a profit from the sale of unsafe products: One participant recognized the financial burden of unsafe products: “…with my first son I had all that stuff, man, sides up and bumpers. Man it was expensive….”

Current advertising media were also perceived as sending conflicting message to fathers by portraying them in unsafe sleeping situations with infants. One participant asked, “Am I supposed to fall asleep with the baby on the couch and get a picture like this? I think a lot of dads don’t really know what they are supposed to be doing the first few months.”

Participants also cited inconvenience as a barrier to safe sleep practices. Consistent co-sleeping participants believed that being near their infant made it easier for infant care: “…now I can tend to that baby when they wake. I’m here, I ain’t got to a deep sleep.” Another participant stated that the child may start out in a crib but that when repeated disruptions to restful sleep is a precipitating factor to co-sleeping: “it’s like nails on a blackboard when she cries…we kinda tried things out and, usually, things don’t last more than an hour and then we are back to co-sleeping.”

Some participants perceived that safety was actually increased by having the child in the bed with adults: “I just feel a lot better if you have them in bed with Daddy than the crib.” One participant expressed that the child’s mother was afraid of using a crib saying, “…other than a good visual on the child…like kids be climbing on the crib, heads getting stuck between the bars, things of that nature, so we just cut all through that.” Some participants occasionally co-slept if their child was breastfeeding or teething.

Personal financial constraints were not mentioned explicitly as barriers, but were implied. No participant cited finances as a barrier for separate sleeping areas or as a justification for co-sleeping. While discussing the use of non-safe products in the crib (e.g., bumper pads, blankets, and stuffed toys) several participants recognized the financial and safety benefits of not using unsafe products in the crib. “Save money don’t buy all that stuff. Put the baby on the back and you’re good” stated one participant. Another stated a similar belief: “Well, it’s actually a lot cheaper to do the safe thing. Don’t have to get…bumpers and the blankets and pillows.” The need for distribution of a Pack-N-Play to households with a child was equated with other public assistance programs stated one participant, “…like DHS applying for food stamps or something like that.”

### Facilitators

Despite multiple barriers to practicing safe sleep, some participants were engaging in safe behaviors. Positive influences included parental agreement regarding safe sleep, family and friends who provided correct information and support of safe behaviors, and prior exposure to safe sleep messaging via personal research or information provided by a medical professional.

If participants had previous knowledge of safe sleep behaviors, they mainly acquired that knowledge from a doctor’s office, a hospital during infant delivery, or some other medical professional or setting such as prenatal or safety classes. One participant stated that, “Every nurse and every pediatrician that I’ve come in contact since he was born was like make sure you put him on his back, no animals or nothing in the crib.” Some caregivers cited previous experience with raising older children that reinforced the practice of safe sleep. The more experienced parents’ sense of perceived susceptibility to sleep-related deaths also may have been higher: They acknowledged the risks of unsafe sleep behaviors and understood if they engaged in those behaviors, their child could be harmed: “…I made a bunch of mistakes as far as safety, sleeping…I tried my best to correct some things that might still let slide….” Another participant qualified co-sleeping as safe under certain conditions: “Oh, you can roll over on them, all them things, but you gotta be a light sleeper. It’s unsafe, but it is safe at the same time.” Some caregivers knew someone whose child died from unsafe sleep, and learned from that situation to keep their child safe.

Many participants emphasized that safe sleep was easily practiced and that doing so is less expensive than buying extraneous toys, blankets, and other objects that could harm sleeping infants. Some participants even noted that if parents don’t have a safe sleep surface, social services may provide them.

### Messaging suggestions

Participants were asked to identify suggestions for future infant safe sleep messages targeted to male caregivers. Overwhelmingly, participants wanted the content and tone of safe sleep messages to be factual, brief, and serious. Statistics and statement of facts regarding national and local death rates were cited as influential in both promoting a health behavior change or maintaining safe sleep. Many participants supported this approach: “Help(s) seeing the numbers. I think numbers are what people pay attention to.” Some participants wanted a humorous rather than serious tone, but most opted for an emotionally-targeted angle, with several recommendations for focusing on the safeguarding role of male caregivers: “this is your little one and you’re the dad…you’ve got to be make sure this house is protected.” Some suggested hearing personal stories by male caregivers who have been affected negatively by unsafe sleep. The majority of participants emphasized that they were involved in child care since birth and that messages should recognize that. For example, some participants suggested emphasizing messages that show male caregivers taking as much pride in their child’s sleep behavior as other important activities including those they have a passion or hobby in (hunting, sports, and mechanics): “My thing… (is) that every ad is like oh dads you don’t play a role until it’s time for sports. Moms got it until it’s time to throw a ball.” The participants strongly advocated for positive images of male caregivers promoting safety rather than as unskilled in child care: “I can tell you one thing I don’t wanna see …I don’t wanna see things where dad’s an idiot; a bumbling idiot.”

Regarding delivery method for safe sleep messages, participants emphasized a desire for quick communication methods including billboards, posters, commercials, and social media. Options for media targeting male consumers, including internet radio, video games, phone apps, and sports venues were also endorsed. Locations for messages primarily included obstetrician and pediatrician’s offices and hospital delivery and discharge. Many stated that they, “…would be most likely to listen to stuff and pay attention to stuff in a pediatrician office. The kind of feeling of oh here is where they tell you to be careful.” Other suggestions for venues included baby supply stores, safety classes, and word-of-mouth.

## Discussion

Sudden Infant Death Syndrome (SIDS) and suffocation account for more than half of all Sudden Unexpected Infant Deaths and are leading causes of deaths in infants ages 28 days to 1 year in the United States (National Center for Health Statistics 2014). Although the incidence of SIDS has decreased since 2008, rates of infant death from Accidental Suffocation and Strangulation in Bed (ASSB) have increased (CDC/NCHS 2015). This pattern may be due to the shift in terminology from SIDS to ASSB when detailed investigations of infant death scenes are conducted, appropriately identifying infant sleep environment as a contributing or causative factor to death.

Universal adoption of risk reduction strategies, including supine sleep position and safe sleep environment, is critical for prevention, but adoption among certain high risk groups is low. For example, extensive literature suggests that African American mothers know, but do not practice infant safe sleep recommendations due to either false safety perceptions or perceived comfort of the infant (Khanom et al. 2013). Barriers and facilitators to compliance with safe sleep recommendations among fathers and male caregivers of infants is less well studied.

Our study found that knowledge of safe sleep recommendations varied among participants. Placing the infant on its back for sleep, which has been promoted for twenty-five years, was common knowledge. Knowledge of other recommendations, such as the use of sleep sacks and separate sleep spaces, was also reported although not consistently practiced. Convenience was commonly reported as a mitigating factor to knowledge, especially for intermittent and naptime sleep. Even fathers who described stronger and more sustained compliance for nighttime sleep admitted that convenience could, at times, influence their behavior. Clinicians and health educators should emphasize to all caregivers that duration is not a predictor of risk for sleep-related deaths and all sleep should be consistent with recommendations.

Our study also found that personal beliefs and cultural norms regarding co-sleeping were stronger barriers than a lack of knowledge. A lack of perception of risk for an infant dying of a sleep-related death was an underlying theme regarding co-sleeping. Many believed that co-sleeping improved the parent-child relationship and parental caretaking. Some participants believed they could reduce co-sleeping risks by controlling or modifying their own sleep behaviors. There was also a belief that co-sleeping-related deaths were out of the control of parents and were acts of fate or God. Participants did not recognize these deaths injury-related deaths. Including education on how suffocation occurs in co-sleeping messages may strengthen fathers’ confidence in their ability to protect their infants, much like car seat technicians use crash dynamics to educate parents about the protective qualities of a car seat.

Fathers and male caregivers felt strongly that safe sleep messaging should appeal to their sense of responsibility as the protector of a child. They believed that the stereotype of a “doofus” dad, cartoon characters, and father’s only being involved with an older child during a gender-focused activity (e.g. sports) are messages that undervalue both quality and quantity of male caregiving roles in infancy. Delivery of these messages should be in a straightforward style with facts and evidence and contain emotional cues to the male parenting role. They preferred dissemination strategies that were already in their normal life activities, such as point-of-purchase education in retail stores and in packaging of baby-related products and typical gathering locations for males, such as sports venues. Clinicians, pre-natal educators, and parenting educators were acceptable sources of information, although numerous participants acknowledged that accurate information is not consistent across these disciplines.

There were limitations to our study. Recruitment was very challenging and we did not achieve the sample size that was planned. In addition, our reliance on assistance from local gatekeepers for recruitment may have resulted in some participants being uninformed about the purpose of the focus groups, leading to less engagement from some participants. The original promotional flyer featured a cartoon drawing of a male caregiver with a young child. After hearing participants’ preferences regarding misrepresented images of fathers and male caregivers, the image on print materials was changed.

## Conclusions

Male caregivers of infants had been exposed to safe sleep messages but adoption of recommended risk reduction strategies was variable, especially with daytime sleep. They endorsed the need for more information about safe sleep directed towards male caregivers, and preferred messages that recognize their role in child care during infancy, representing them in a positive manner as having a critical role in infant safety.

## Abbreviations

AAP: American Academy of Pediatrics
ASSB: Accidental suffocation and strangulation in bed
FG: Focus group
HBM: Health Belief Model
SIDS: Sudden Infant Death Syndrome
SUID: Sudden Unexplained Infant Death

## References

[CR1] Adams LE, Aitken ME, Mullins SH, Miller BK, Graham J (2013). Barriers and facilitators to all-terrain vehicle helmet use. J Trauma Acute Surg.

[CR2] Ball HL, Hooker E, Kelly PJ (2000). Parent-infant co-sleeping: Father’s roles and perspectives. Infant Child Dev.

[CR3] CDC/NCHS (2015). National Vital Statistics System. Compressed mortality file.

[CR4] Goodlin-Jones BL, Burnham MM, Gaylor EE, Anders TF (2001). Night waking, sleep-wake organization, and self-soothing in the first year of life. J Dev Behav Pediatr.

[CR5] Herman S, Adkins M, Moon RY (2015). Knowledge and beliefs of African-American and American Indian parents and supporters about infant safe sleep. J Commun Health.

[CR6] Holtz B, Smock A, Reyes-Gastelum D (2015). Connected motherhood: social support for moms and moms-to-be on Facebook. Telemed Journal and e-Health.

[CR7] Keener MA, Zeanah CH, Anders TF (1988). Infant temperament, sleep organizations, and nighttime parental interventions. Pediatrics.

[CR8] Khanom A, Hills RA, Brophy S, Morgan K, Rapport F, Lyons R (2013). Mothers’ perspectives on the delivery of childhood injury messages: a qualitative study from the growing up in Wales, environments for health living study. BioMed Central.

[CR9] Millikovsky-Ayalon M, Atzaba-Poria N, Meiri G (2015). The role of the father in child sleep disturbance: child, parent, and parent-child relationship. Infant Mental Health Journal.

[CR10] Moon RY, Oden RP, Joyner BL, Ajao TI (2010). Qualitative analysis of beliefs and perceptions about sudden infant death syndrome (SIDS) among African-American mothers: implications for safe sleep recommendations. J Pediatr.

[CR11] National Center for Health Statistics (2014). National Vital Statistics Reports.

[CR12] Poag S, Susswein M, Baker T. Prevention and Early Intervention: Safe Sleep Final Report. SUMA Social Marketing for Texas Department of Family and Protective Services. 2016.

[CR13] Research Ware Inc (1988). HyperRESEARCH.

[CR14] Rosenstock IM, Strecher VJ, Becker MH (1988). Social learning theory and the health belief model. Health Educ Q.

[CR15] Sadeh A, Tikotzky L, Scher A (2010). Parenting and infant sleep. Sleep Medicine Review.

[CR16] Tikotzky L, Sadeh A, Glickman-Gavrieli T (2011). Infant sleep and paternal involvement in infant caregiving during the first six months of life. J Pediatr Psychol.

[CR17] Tikotzky L, Sadeh A, Volkovich E, Manber R, Meiri G, Shahar G (2015). Infant sleep development from 3 to 6 months postpartum: links with maternal and paternal involvement. Monograph Society for Research in Child Dev.

[CR18] Tong A, Sainsbury P, Craig J (2007). Consolidated criteria for reporting qualitative research (COREQ): a 32-item checklist for interviews and focus groups. Int J Qual Health Care.

